# Exogenous treatment with melatonin enhances waterlogging tolerance of kiwifruit plants

**DOI:** 10.3389/fpls.2022.1081787

**Published:** 2022-12-09

**Authors:** Liuqing Huo, Hujing Wang, Qi Wang, Yongbin Gao, Kai Xu, Xuepeng Sun

**Affiliations:** Collaborative Innovation Center for Efficient and Green Production of Agriculture in Mountainous Areas of Zhejiang Province, Key Laboratory of Quality and Safety Control for Subtropical Fruit and Vegetable, Ministry of Agriculture and Rural Affairs, College of Horticulture Science, Zhejiang A&F University, Hangzhou, Zhejiang, China

**Keywords:** waterlogging, kiwifruit, melatonin, amino acid, GABA

## Abstract

Waterlogging stress has an enormous negative impact on the kiwifruit yield and quality. The protective role of exogenous melatonin on water stress has been widely studied, especially in drought stress. However, the research on melatonin-induced waterlogging tolerance is scarce. Here, we found that treatment with exogenous melatonin could effectively alleviate the damage on kiwifruit plants in response to waterlogging treatment. This was accompanied by higher antioxidant activity and lower ROS accumulation in kiwifruit roots during stress period. The detection of changes in amino acid levels of kiwifruit roots during waterlogging stress showed a possible interaction between melatonin and amino acid metabolism, which promoted the tolerance of kiwifruit plants to waterlogging. The higher levels of GABA and Pro in the roots of melatonin-treated kiwifruit plants partly contributed to their improved waterlogging tolerance. In addition, some plant hormones were also involved in the melatonin-mediated waterlogging tolerance, such as the enhancement of ACC accumulation. This study discussed the melatonin-mediated water stress tolerance of plants from the perspective of amino acid metabolism for the first time.

## Introduction

Water stress (waterlogging and drought) is becoming one of the most common abiotic stresses in the agriculture system, mainly due to the frequent occurrence of global warming, rainfall disparity, and poor drainage (Stuart et al., 2011; Hussain et al., 2018). Waterlogging stress often takes place in tropical and subtropical regions, especially during the rain seasons (Phukan et al., 2016). It was estimated that over 10% of the agricultural land is under the threat of waterlogging stress, and in severe cases waterlogging can result in nearly 40-80% of the crop yield losses (Kreuzwieser and Rennenberg, 2014; Shabala et al., 2014). With the increased duration and intensity of extreme precipitation, waterlogging stress may be more severe in the future, which seriously threatens global food security (Li et al., 2022).

The excessive waterlogging blocked air exchange between soil and atmosphere, resulting in restricted oxygen availability for plant roots, thereby suppressing roots respiration, decreasing root activity, and causing root damage (Pan et al., 2021; van Veen et al., 2014). Another key impact of waterlogging stress is the excessive generation of reactive oxygen species (ROS), which directly and indirectly damages the cell membrane, proteins, and macromolecules (Zhang et al., 2017). These unproperly disposed ROS finally cause oxidative damage to roots and leaves, impairing the water uptake of plants, accompanied by leaf chlorosis and wilting (Hossain et al., 2009). In addition, during waterlogging stress, the leaf stomata closure is generally observed in plants, whereas chlorophyll degradation and leaf senescence adversely affect gas exchange and photosynthetic rate, which ultimately lead to a decline in the quantity and quality of crops (Arbona et al., 2010; Nguyen et al., 2012).

Indeed, plants have evolved various strategies to defend themselves against waterlogging stress. Some plants showed adaptive responses such as altering their morphologies, including aerenchyma formation, stem elongation, and the formation of adventitious roots, which is known as the low oxygen escape syndrome (Kuroh et al., 2018; Qi et al., 2020). Moreover, some plants use the quiescence strategy to alleviate the adverse effect of waterlogging stress, including the down-regulation of a suite of metabolic pathways, low growth rate, and protection against oxidative stress (Geigenberger, 2003; Garssen et al., 2015). A series of antioxidant mechanisms are activated to help plants to maintain proper cellular ROS levels under waterlogging stress. These include antioxidant enzymes, such as superoxide dismutase (SOD) and peroxidase (POD), and small molecule antioxidants (Mittler et al., 2011). In particular, as a unique antioxidant, melatonin (N-acetyl-5-methoxytryptamine) is proved to be a potent free radical scavenger and regulate various plant responses to environmental disorders (Back et al., 2016; Arnao and Hernández-Ruiz, 2019). Melatonin has been widely proved to be protective in diverse plant species under a variety of abiotic and biotic stresses (Gao et al., 2022), such as cold stress (Bajwa et al., 2014), oxidative stress (Wang et al., 2015), drought stress (Liang et al., 2019) and et al. However, studies on the roles of melatonin in waterlogging tolerance of plants are rare (Moustafa-Farag et al., 2020).

The metabolic acclimation to waterlogging stress has also been widely studied on whole seedlings or roots of plants, in which the primary nitrogen metabolism is proved to be one of the important adaptive responses in plants (Bailey-Serres et al., 2012; Oliveira and Sodek, 2013). As the important basis for synthesis of nitrogenous substances, amino acid metabolism is an important adjustment strategy of plants in responding to waterlogging stress (Batista-Silva et al., 2019). For example, an increase in the accumulation of γ-aminobutyrate (GABA) and alanine (Ala) was always observed in plants under waterlogging stress (Drew, 1997). Recent research found that roots responded more strongly to waterlogging stress while high amounts of GABA and lactate accumulation were detected in roots other than shoots; Moreover, the Ala accumulation was detected in both organs (Mustroph et al., 2014). Change in the level of plant hormones is another important strategy for plants to adapt to waterlogging stress (Dat et al., 2004). For example, previous researches found that waterlogging stress induced a significant depletion in the endogenous ABA levels in submerged tissues of various plant species, such as the model plant *Arabidopsis thaliana* (Liu et al., 2005) and the important perennial fruit tree citrus (Arbona et al., 2017). In addition, a fast cellular accumulation of the gaseous plant hormone ethylene has been reported in submerged plants, which act as a reliable component of the waterlogging sensing mechanism and crosstalk with other hormones for surviving (Kreuzwieser and Rennenberg, 2014). Particularly, endogenous ethylene production is at the basis of the induction and formation of aerenchyma cells (Fukao and Bailey-Serres, 2008).

Kiwifruits are popular for its unique flavor and high vitamin C content; however, the waterlogging problem severely decreased the annual yield of kiwifruit plants in their main planting areas (Fukao and Bailey-Serres, 2008). The use of melatonin in various field trials has validated that as an eco-friendly agrochemical, it has great potential for applying in horticultural industry to solve agricultural challenges (Gao et al., 2022). To date, the application of melatonin in agriculture systems to deal with waterlogging problems has been understudied. Here, we found that the pre-irrigation of 100 μM melatonin in kiwifruit plants could effectively alleviate the waterlogging damage on them. In addition to the antioxidant system, the amino acid metabolic pathways are also involved in the melatonin-mediated waterlogging tolerance of kiwifruit plants. Particularly, the GABA and proline (Pro) synthesis in kiwifruit roots was enhanced by melatonin treatment to encounter waterlogging stress. The 1-aminocyclopropane-1-carboxylate (ACC) accumulation in kiwifruit roots might also participate in the melatonin-mediated waterlogging tolerance. This study provides the evidence for the links between melatonin and amino acid metabolic systems in the stress tolerance of plants.

## Materials and methods

### Plant materials and treatments

Tissue-cultured plants of Actinidia chinensis var. deliciosa cv. Qinmei were initially grown on MS agar media containing 2.0 mg/L ZT and 0.1 mg/L IBA. They were cultured under conditions of 25°C, 60 μmol/m^2^/s and a 14-h photoperiod. After rooting on 1/2 MS agar media containing 0.5 mg/L IBA for 40 days, the plantlets were transferred to black plastic pots (8 × 8 cm) containing a mixture of loam/perlite (1:1, v:v). After 40 d of adaptation in the growth chamber, the plants were moved to larger plastic pots (30 × 18 cm) filled with a mixture of soil/sand/organic matter (5:1:1, v:v:v) and grown in the glasshouse. They were watered with tap water or 1/2 Hoagland nutrient solution (Huo et al., 2021) alternately every three days.

After two months of growth under above conditions, healthy and uniformly sized plants were chosen for screening the optimal concentration of melatonin treatment: plants were fully irrigated with 300 ml 0, 10, 50, 100, 200, or 300 μM melatonin solution to the roots for 2 times in a three-day interval and then were waterlogged. After 9 days of waterlogging treatment, the plant roots were collected to measure root activity. Finally, we selected 100 μM as the melatonin concentration for downstream experiments.

The treatments were subdivided into four groups as follows: (1) control (CK), plants were well-watered during the whole experimental time; (2) melatonin treatment (MT): plants were treated with 100 µM melatonin solution for 2 times in a three-day interval, then been well-watered for 9 days; (3) waterlogging treatment (WL): plants were well-watered for 6 days, and subsequently been waterlogged for 9 days; and (4) melatonin and waterlogging treatment (MT+WL): plants were treated with 100 µM melatonin solution for 2 times in a three-day interval, and then been waterlogged for 9 days. Each treatment included 20 pots of plants and was repeated three times. To apply waterlogging treatments, three potted plants were placed in a plastic container (100 × 35 × 30 cm) filled with tap water, and the water level was continuously maintained at 4-5 cm above the soil surface. The day before waterlogging was designed as 0 day, and the third through fifth fully matured leaves from the base of the stems and the roots were sampled from all groups at 0, 3, 6, and 9 days of the experiment. For samples mentioned above, three biological replicates were prepared with each collected from three plants. The samples were stored at −80°C after being frozen quickly in liquid nitrogen until use.

### Evaluation of stress tolerance

The relative electrolyte leakage of the leaves and roots was determined and calculated according to a previously described method with a minor modification (Dionisio-Sese and Tobita, 1998). Briefly, 100 mg fresh plant tissue samples were cut into 5 mm length and placed in test tubes containing 10 ml distilled deionized water. The tubes were covered with plastic caps and placed in a water bath maintained at the constant temperature of 25 °C. 4 h later, the initial electrical conductivity of the distilled deionized water and the medium in test tubes was measured using an electrical conductivity meter (CM-115, Kyoto Electronics, Kyoto, Japan). Then, the samples were autoclaved at 100°C for 15 min to completely kill the tissues and release all electrolytes. Afterwards, they were cooled for 4 h to 25°C and the final electrical conductivity was measured.

Levels of malondialdehyde (MDA), H_2_O_2_, and superoxide radical (O_2_^−^) and the activities of superoxide dismutase (SOD) and peroxidase (POD) were determined using detection kits (Suzhou Comin Biotechnology Co., Ltd, Suzhou, China) following the manufacturer’s instructions. The triphenyl tetrazolium chloride method was applied to monitor root activity, which was defined as the reductive intensity.

### Evaluation of photosynthetic characteristics

The net photosynthesis rate (Pn), intercellular CO_2_ concentration (Ci), and stomatal conductance (Gs) were monitored by a LI-6400XT portable photosynthesis system (LI-COR, Huntington Beach, CA, USA). All measurements were taken between 9:00 and 11:00 a.m. at 1000 μmol photons m^−2^ s^−1^ and a constant airflow rate of 500 μmol s^−1^. The concentration of cuvette CO_2_ was set at 400 ± 5 cm^3^ m^−3^, and the temperature was 28 ± 2°C. Data were collected from the fully expanded, fully light-exposed leaves at the same position of three plants.

### Measurement of amino acids

Amino acids (AAs), such as GABA and Pro, were extracted and measured as described previously (Huo et al., 2020), with a minor modification. Briefly, 100 mg of frozen root sample was extracted in 1 ml 50% ethanol (including 0.1 M HCl). After centrifuging at 13,000 g for 10 min, the liquid supernatant was filtered through a 0.22-μm filter and the filtered supernatant was diluted 20 times using methanol to analyze the metabolites. The liquid chromatography-mass spectrometry system (LC-MS, LC: AC, ExionLC; MS: Q-trap5500, AB Sciex Pret. Ltd., Washington, USA) equipped with an Inertsil ODS-4 C18 column (4.6 × 250 mm, 5 mm) was used at a flow rate of 0.3 ml/min, and 10 μL was used as sample injection volume. The solvent system consisted of water containing 0.1% (v/v) formic acid (A) and acetonitrile (B). Data were quantified by comparing the peak surface areas with those obtained using standard AAs (Sigma, St. Louis, MO, USA).

### Measurement of phytohormones

ABA, ACC and SA were extracted as described previously (Huo et al., 2020) and measured using the LC-MS system. Briefly, 200 mg of frozen root sample was extracted in 1 ml solvent (methanol: isopropanol: acetic acid= 20: 79: 1; v: v: v). After shaking for 1 h at 4°C and centrifugation, the supernatant was filtered through a 0.22-μm filter for analysis using the same system described above but equipped with the InertSustain AQ-C18 column (4.6 × 150 mm, 5 μm) at a flow rate of 0.5 ml/min. The solvent system consisted of methanol (A) and water containing 0.1% (v/v) formic acid (B).

Melatonin was quantified as described by Zhou et al. (2022), with some modifications. Briefly, 500 mg of frozen root sample was suspended in 5 ml methanol. After shaking overnight at −20°C and centrifugation, the supernatants were collected for drying under nitrogen gas. Then, the dried extracts were dissolved in 200 µl 80% methanol and filtered through a 0.22-µm filter membrane. The analysis system was the same as described above.

### Statistical analysis

SPSS 22.0 software was used for statistical data analysis. All experimental data were subjected to one-way analysis of variance, and the statistical differences were calculated by Tukey’s multiple range test (p < 0.05). Values were presented as means ± SEs (standard errors) of at least three biological replicates.

## Results

### Exogenous melatonin improves the waterlogging tolerance of kiwifruit plants

**Figure 1:**
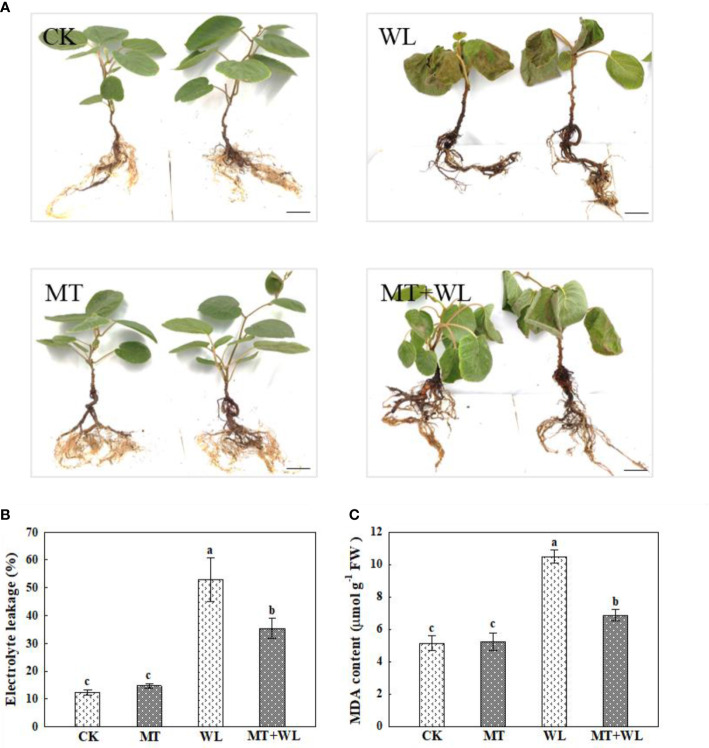
Exogenous melatonin alleviated the waterlogging damage on kiwifruit plants. **(A)** Phenotypes of kiwifruit plants in control (CK), melatonin treatment (MT), waterlogging treatment (WL), and melatonin and waterlogging treatment (MT+WL) groups, and the plants in WL and MT+WL groups were being waterlogged for 9 days. Bars: 5 cm. **(B)** Relative electrolyte leakage (REL) and **(C)** malondialdehyde (MDA) concentration of plants in CK, MT, WL, and MT+WL groups. Data are shown as the means of three replicates with SEs. Different letters indicate significant differences between treatments, according to one-way ANOVA and Tukey’s multiple range test (P < 0.05).

To investigate the possible effect of exogenous melatonin on kiwifruit plants in response to waterlogging stress, we first pretreated the plants with different concentrations of melatonin, and then measured their root activity after waterlogging treatment. The results showed that melatonin treatment effectively alleviated waterlogging damage on root activity of kiwifruit plants (**Table 1**). According to our evaluation, the 100 μM melatonin pretreatment performed better than 10 or 50 μM, meanwhile it was more economical than 200 or 300 μM on the basis of similar effect on waterlogging tolerance of kiwifruit plants. Then we applied 100 μM melatonin solution to irrigate designated plants before inducing waterlogging stress. Under normal-watered conditions, the root morphology did not differ apparently between kiwifruit plants treated with (MT) or without (CK) melatonin. After waterlogging stress (WL), the leaves of kiwifruit plants in both WL and MT+WL groups wilted severely and brown patches were observed. Moreover, while the lower leaves of plants in the WL group appeared serious shedding due to waterlogging stress, plants received melatonin pretreatment showed lighter stress symptoms and many mature leaves remained green and vigorous (**Figure 1A**).

**Table 1:** Effects of melatonin pretreatment (10~300 μM) on root activity of waterlogged kiwifruit plants.

Treatment group	TTC reductive intensity (μg g^-1^ FW h^-1^)
**CK**	153.08 ± 8.85 a
**WL**	86.05 ± 12.05 d
**WL + 10 μM**	100.30 ± 6.45 cd
**WL + 50 μM**	101.98 ± 8.34 cd
**WL + 100 μM**	120.81 ± 7.70 b
**WL + 200 μM**	115.34 ± 6.10 bc
**WL + 300 μM**	114.49 ± 7.84 bc

All data are means ± SE of three replicates. Values not followed by the same letter indicate significant differences between treatments, according to one-way ANOVA followed by Tukey’s multiple range test (P <0.05).

The relative electrolyte leakage and malondialdehyde (MDA) content are typical parameters for assessing stress tolerance of plants (Dionisio-Sese and Tobita, 1998; Huo et al., 2020). The electrolyte leakage of plant cell is a basic indicator of cell membrane permeability, for the value positively correlates with the severity of membrane destruction. There was no significant difference between the electrolyte leakage of CK and MT plants under normal-watered condition. After waterlogging treatment, while the electrolyte leakage being significantly increased by 4.3 times in WL plants compared to CK plants, the increment was only 2.9 times in MT+WL plants when compared with MT plants (**Figure 1B**). Moreover, the levels of MDA in plant cells can reflect the extent of lipid peroxidation, which is an indicator of cell damage. The data showed that while the MDA content was increased in waterlogging-stressed leaves when compared with the control, exogenous melatonin alleviated that increment (**Figure 1C**). These data demonstrated that exogenous melatonin alleviated the waterlogging damage on kiwifruit plants.

### Effects of exogenous melatonin on the photosynthetic capacity of kiwifruit plants under waterlogging stress

**Figure 2:**
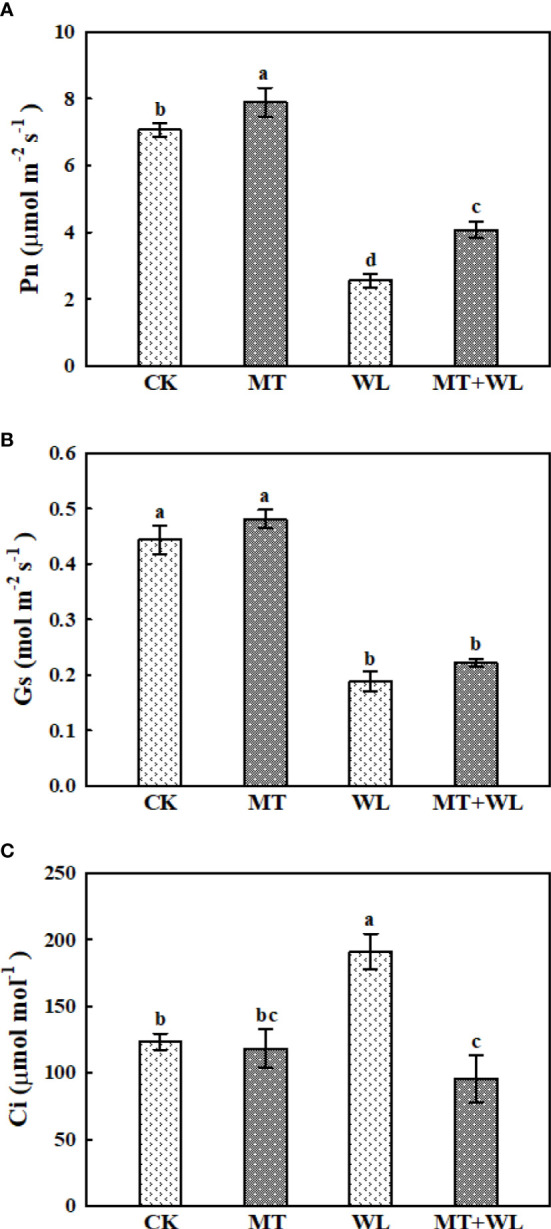
Exogenous melatonin alleviated the waterlogging damage on the photosynthetic ability of kiwifruit plants. Changes in the **(A)** net photosynthesis rate (Pn), **(B)** stomatal conductance (Gs) and **(C)** intercellular CO_2_ concentration (Ci) were determined after 6 days of waterlogging treatment. Data are shown as the means of three replicates with SEs. Different letters indicate significant differences between treatments, according to one-way ANOVA and Tukey’s multiple range test (P < 0.05).

Under waterlogging stress, the efficiency of photosynthetic system was seriously inhibited due to leaf necrosis and stomatal behavior. To examine the effect of melatonin on photosynthesis, we detected the gas exchange parameters after 6 days of waterlogging treatment. As shown in **Figure 2A**, Pn decreased in both WL and MT + WL group, which indicates the suppressed assimilation efficiency of CO_2_, but the rates were significantly higher in melatonin-pretreated plants than in non-pretreated plants. The Gs of kiwifruit plants also decreased under waterlogging stress, and it was marginally but not significantly higher in melatonin-pretreated plants on the 6th day (**Figure 2B**). Moreover, there was a substantial increase in the Ci value of non-pretreated plants after 6 d of waterlogging stress, but that increment was not presented in the melatonin-pretreated plants (**Figure 2C**). These results demonstrated that while the photosynthetic ability of kiwifruit plants was damaged by waterlogging stress, exogenous melatonin effectively alleviated that pressure.

### Exogenous melatonin decreased ROS damage on the roots of kiwifruit plants under waterlogging stress

**Figure 3:**
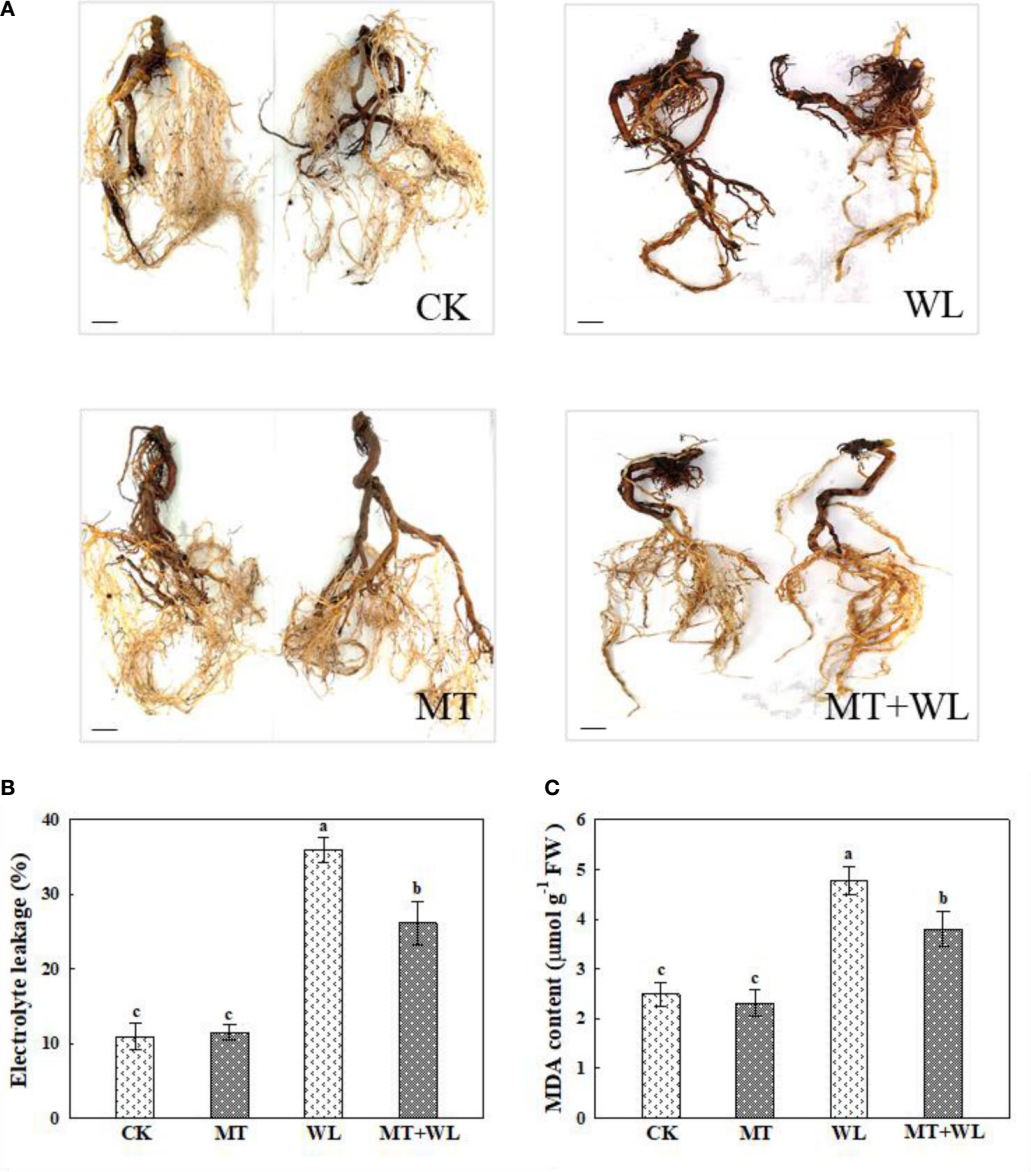
Exogenous melatonin alleviated the waterlogging damage on the roots of kiwifruit plants. **(A)** Phenotypes of the roots of kiwifruit plants in CK, MT, WL, and MT+WL groups, and the plants in WL and MT+WL groups were being waterlogged for 9 days. Bars: 5 cm. **(B)** Relative electrolyte leakage (REL) and **(C)** malondialdehyde (MDA) concentration in the roots of CK, MT, WL, and MT+WL plants. Data are shown as the means of three replicates with SEs. Different letters indicate significant differences between treatments, according to one-way ANOVA and Tukey’s multiple range test (P < 0.05).

As shown in **Figure 3A**, the waterlogging damage on the roots of kiwifruit plants was remarkably relieved by melatonin treatment, represented by more vigorous and white roots on the plants in melatonin-treated groups than in non-pretreated plants after waterlogging stress. These results were further demonstrated by the relative electrolyte leakage and MDA measurements of plant roots, which were increased significantly due to the injury caused by waterlogging stress, but they were still much lower in MT+WL plants compared with that in WL plants (**Figures 3B, C**).

**Figure 4:**
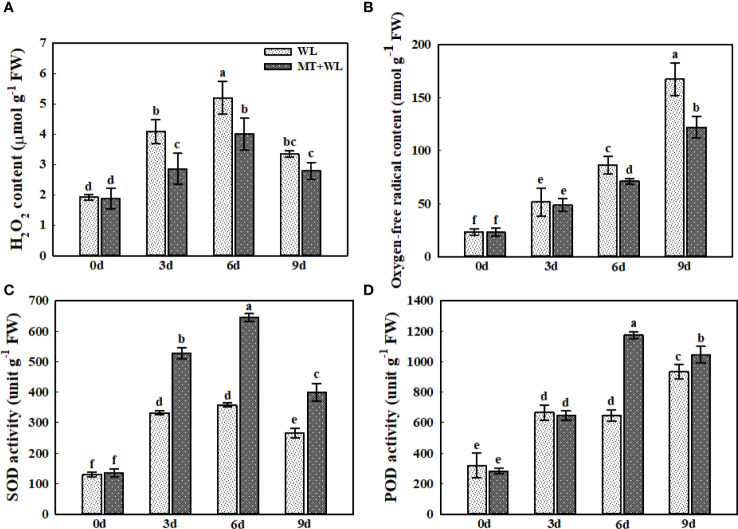
Exogenous melatonin alleviated the ROS damage in the roots of kiwifruit plants under waterlogging stress. **(A)** H_2_O_2_ and **(B)** O_2_^−^ concentrations in the roots of CK, MT, WL, and MT+WL plants. Activities of **(C)** superoxide dismutase (SOD) and **(D)** peroxisome (POD) in the roots of CK, MT, WL, and MT+WL plants. Data are shown as the means of three replicates with SEs. Different letters indicate significant differences between treatments, according to one-way ANOVA and Tukey’s multiple range test (P < 0.05).

The stress-induced ROS accumulation leads to oxidative stress by disrupting cytomembranes and cell components, ultimately affecting plant growth. Here, the measurements of H_2_O_2_ and O_2_^−^ levels showed that they were both increased in plant roots during the treatment (**Figures 4A, B**). The H_2_O_2_ content reached the peak after 6 d of treatment and then declined at 9 d, while the O_2_^−^ content still showed an upward trend after 9 d of treatment. Moreover, the H_2_O_2_ levels were significantly higher in the roots of WL plants than in MT+WL plants from day 3 of treatment, and this difference of O_2_^−^ contents between the two groups became obviously clear after 6 d of treatment. We measured the activity of SOD and POD enzymes throughout the treatment, since SOD converts destructive O_2_^−^ into H_2_O_2_ and POD can break H_2_O_2_ down immediately into water (**Figures 4C, D**). The results showed that both SOD and POD activities were induced by waterlogging stress in kiwifruit roots. The SOD activity in the roots of MT+WL plants were 1.6-times that of WL plants on day 3 and day 6 of treatment. Moreover, the POD activity in the roots of MT+WL plants was rapidly increased on day 6 of treatment, where it was 1.82-times that of WL plants. These results suggested that melatonin treatment promoted the SOD and POD activity in the roots of kiwifruit plants and thus alleviated the ROS damage under waterlogging stress.

### Ala, GABA, Pro metabolism in the roots of kiwifruit plants under waterlogging stress

**Figure 5:**
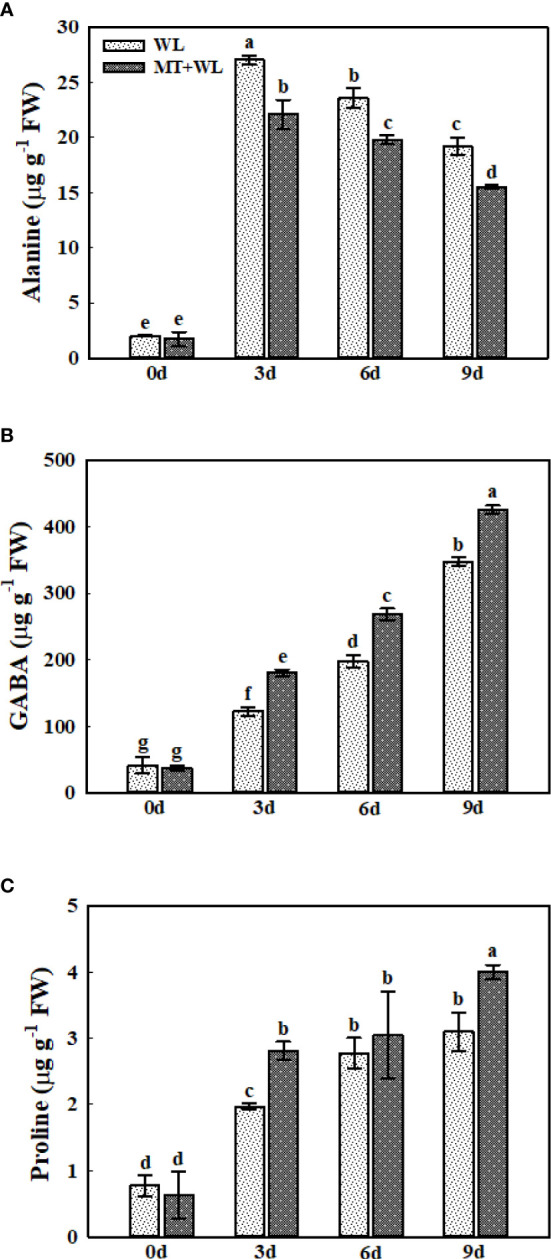
The concentrations of Ala, GABA, and Pro in the roots of kiwifruit plants under waterlogging stress, as measured by LC-MS. **(A)** Ala. **(B)** GABA. **(C)** Pro. Data are shown as the means of three replicates with SEs. Different letters indicate significant differences between treatments, according to one-way ANOVA and Tukey’s multiple range test (P < 0.05).

Previous studies have reported the accumulation of Alanine (Ala) and γ-aminobutyrate (GABA) in plant roots in response to waterlogging stress (Lothier et al., 2020), and Ala accumulation is thought to be a hypoxic biomarker in plants (Planchet et al., 2017). Here, we detected a pronounced increment in Ala and GABA concentration in the roots of kiwifruit plants in both WL and MT+WL groups under waterlogging treatment (**Figures 5A, B**). The Ala content was increased by 13.3 times in WL plants at 3 d of treatment compared to 0 d, and it was gradually declined afterwards, but still consistently higher than samples without waterlogging treatment. Moreover, it was lower in melatonin-treated plants than in non-pretreated plants from the day 3 of treatment (**Figure 5A**). The GABA content in kiwifruit roots was elevated under waterlogging stress throughout the treatments, and it was significantly higher in melatonin-treated plants than in non-pretreated plants from the day 3 (**Figure 5B**). The Pro content followed the same trend as GABA under waterlogging stress. More specifically, in non-pretreated plants, it was 2.54-, 3.58- and 4.01-times that of 0 d on 3, 6 and 9 d of waterlogging treatment, while it was 4.45-, 4.82- and 6.32-times that of 0 d on 3, 6 and 9 d in melatonin-treated plants (**Figure 5C**). These results suggested that melatonin treatment altered the Ala, GABA and Pro metabolism in the roots of kiwifruit plants in response to waterlogging stress.

### Asp, Glu, Arg metabolism in the roots of kiwifruit plants under waterlogging stress

**Figure 6:**
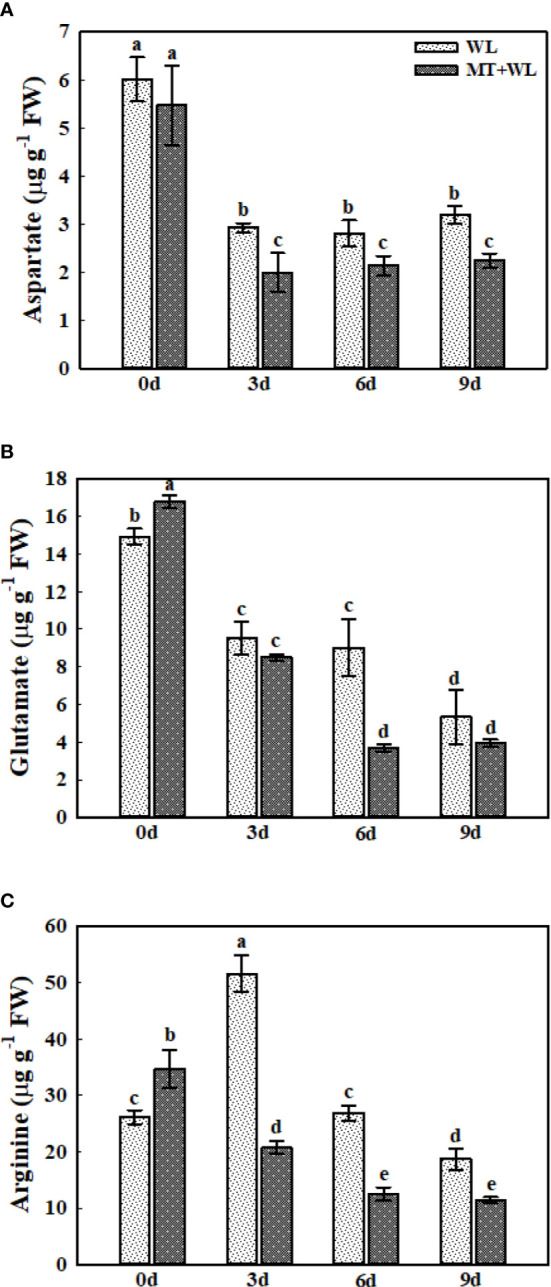
The concentrations of Asp, Glu, and Arg in the roots of kiwifruit plants under waterlogging stress, as measured by LC-MS. **(A)** Asp. **(B)** Glu. **(C)** Arg. Data are shown as the means of three replicates with SEs. Different letters indicate significant differences between treatments, according to one-way ANOVA and Tukey’s multiple range test (P < 0.05).

Aspartate (Asp), glutamate (Glu), and arginine (Arg) are commonly used as nitrogen storage and transport compounds in plants (Hildebrandt, 2018). Here, we analyzed the level of these three amino acids in the roots of kiwifruit plants under waterlogging stress (**Figures 6A–C**). At 3 d of the treatment, there was an obvious decrease of Asp concentration in kiwifruit roots, and the Asp content remained almost unchanged after 3 d. Interestingly, the Asp content was always lower in melatonin-treated plants than in non-pretreated plants from 3 d of treatment (**Figure 6A**). The Glu concentration was gradually declined in the roots during the stress treatments, and it was lower in melatonin-treated plants than in non-pretreated plants from the 6 d of treatment (**Figure 6B**). The Arg content in the roots of WL plants was first increased at 3 d of the treatment, and then declined on 6 d and 9 d, but it was always showing a downward trend in the MT+WL group from the 3 d of the treatment (**Figure 6C**). These results showed that the Asp, Glu and Arg metabolism in the roots of kiwifruit plants was altered by melatonin treatment to encounter waterlogging stress.

### The branched-chain amino acids metabolism in the roots of kiwifruit plants under waterlogging stress

**Figure 7:**
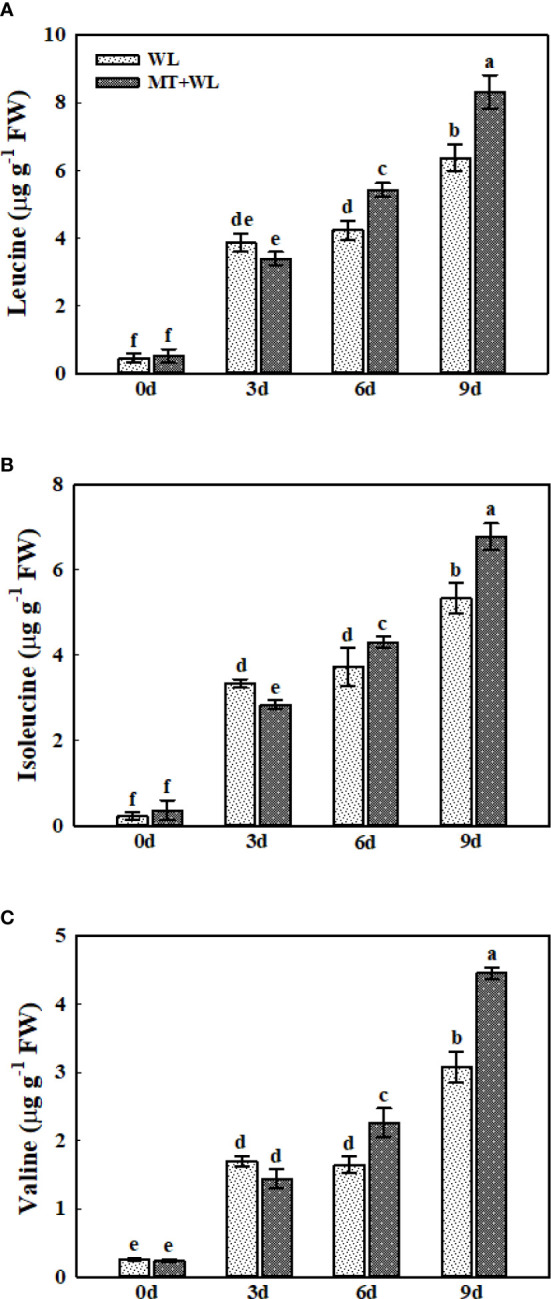
Change to "The concentrations of Leu, Ile, and Val in the roots of kiwifruit plants under waterlogging stress, as measured by LC-MS. **(A)** Leu. **(B)** Ile. **(C)** Val. Data are shown as the means of three replicates with SEs. Different letters indicate significant differences between treatments, according to one-way ANOVA and Tukey’s multiple range test (P < 0.05).

The branched-chain amino acids (BCAAs), including leucine (Leu), isoleucine (Ile), and valine (Val) often show a higher level of accumulation in *Arabidopsis thaliana* and other plant species in response to stress conditions (Sanchez et al., 2010; Huang and Jander, 2017). Here, we detected a pronounced accumulation of these three amino acids in the roots of kiwifruit plants in response to waterlogging stress (**Figures 7A–C**). In non-pretreated plants, the Leu concentration was 8.56-, 9.40- and 14.15-times that of 0 d on 3, 6 and 9 d of waterlogging treatment, while it was 6.60-, 10.53- and 16.17-times respectively in melatonin-treated plants (**Figure 7A**). The levels of Ile followed the same trend, which was a bit higher in melatonin-treated plants than in non-pretreated plants from day 6 (**Figure 7B**). Moreover, the Val concentration was 6.60-, 6.39- and 12.00-times that of 0 d on 3, 6 and 9 d of waterlogging treatment in WL plants, while it was 6.18-, 9.71- and 19.12-times respectively in MT+WL plants (**Figure 7C**). These results showed that waterlogging stress caused a multiple-fold increase in the levels of BCAAs in the roots of kiwifruit plants, and melatonin treatment promoted it in the later stages of stress.

### Plant hormones metabolism in the roots of kiwifruit plants under waterlogging stress

**Figure 8:**
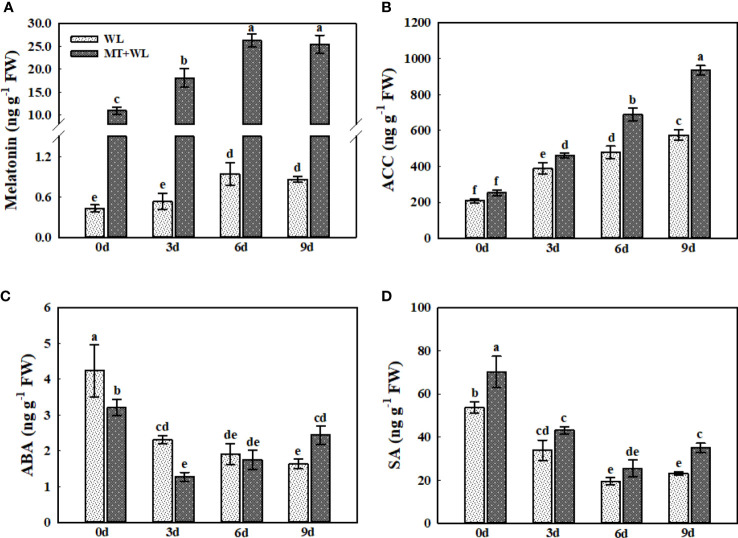
The concentrations of melatonin, ACC, ABA, and SA in the roots of kiwifruit plants under waterlogging stress, as measured by LC-MS. Data are shown as the means of three replicates with SEs. Different letters indicate significant differences between treatments, according to one-way ANOVA and Tukey’s multiple range test (P < 0.05).

Plant hormones are involved in the regulation of waterlogging stress response *via* complex signalling pathways in plants (Benschop et al., 2006; Pan et al., 2021). Here, we analyzed the concentrations of melatonin, ACC (1-aminocyclopropane-1-carboxylate), ABA (abscisic acid), and SA (salicylic acid) in the roots of kiwifruit plants in response to waterlogging stress (**Figures 8A–D**). The measurements of melatonin contents showed that melatonin-treatment promoted it concentrations from 0.4 ng g^-1^ FW to 11 ng g^-1^ FW in kiwifruit roots. The melatonin concentrations in kiwifruit roots were induced by waterlogging stress, which peaked at 6 d of treatment reaching 2.21-times that of 0 d in non-pretreated plants. Interestingly, although there was already sufficiently high concentration of melatonin detected in melatonin-treated groups on 0 d, it was still induced by waterlogging stress in kiwifruit roots, which followed the same trend as in non-pretreated plants, showing 2.39-times that of 0 d at 6 d of treatment (**Figure 8A**). The ACC concentration in kiwifruit roots was enhanced by waterlogging stress throughout the treatment, and it was significantly higher in melatonin-treated plants than in non-pretreated plants from day 3 (**Figure 8B**). The ABA content in kiwifruit roots was down-regulated by waterlogging treatment, and it was lower in melatonin-treated plants than in non-pretreated plants from 0 d to 3 d. Moreover, it showed a certain degree of recovery in MT+WL plants from the day 6, but still reducing in WL group (**Figure 8C**). The SA content in kiwifruit roots was falling down due to the waterlogging stress, but it was always higher in MT+WL plants than in WL plants during the treatments (**Figure 8D**). These results suggested that the balance of plant hormones was changed in kiwifruit roots to encounter waterlogging stress.

## Discussion

As sessile organisms, plants cannot escape from harsh environmental disorders, such as continuous oxygen shortage induced by waterlogging or submergence conditions. Despite this obvious weakness, plants are more tolerant to hypoxia stress than animals, even in species not adapted to submersed soil, which suggest that plants have evolved particular strategies to cope with waterlogging stress (Planchet et al., 2017). Recent studies found that the appropriate metabolism changes are important for plant waterlogging tolerance (Fukao and Bailey-Serres, 2004; Zheng et al., 2017). For example, after a period of waterlogging stress, the plant seedlings changed their metabolism from aerobic to anaerobic respiration, which led to a burst of reactive oxygen species (ROS) generation and resulted in oxidative damages of plant organelles (Xu et al., 2013; Takahashi et al., 2015). The excessive production of ROS in plant roots would disturb their growth and impair the uptake of water and nutrient from soil, therefore disturbing plant gas exchange and photosynthesis (Hossain et al., 2009). The proper ROS scavenging systems and the maintenance of photosynthetic ability are important for plants in response to waterlogging stress. Melatonin has been generally reported to be an effective antioxidant, with important functions in scavenging ROS and regulating plant responses to various environmental stresses (Li et al., 2018; Wu et al., 2020). However, the effects of melatonin in plant physiological and metabolic responses to waterlogging stress have yet to be elucidated (Moustafa-Farag et al., 2020).

The kiwifruit plants are always intolerant to waterlogging stress as the root system is fleshy and shallow. In many kiwifruit planting regions, especially in Southeast China, excessive rainfall in summer rainy season generally causes huge damage to kiwifruit gross yield (Liu et al., 2022). Therefore, studying the waterlogging response mechanisms of kiwifruit is urgent and has important implications for horticultural industry. Previous studies have found that exogenous melatonin could induce the tolerance of kiwifruits to drought stress and alleviate the heat-related damage, mainly through the antioxidant pathways (Liang et al., 2018; Xia et al., 2020). Here, we pre-irrigated the kiwifruit plants with melatonin solution to the roots, and found that exogenous melatonin pretreatment effectively alleviated the decline in the root activity of kiwifruit plants caused by waterlogging stress. We selected 100 μM melatonin as the exogenous concentration to investigate the potential metabolic role of exogenous melatonin on kiwifruit plants under waterlogging stress. The exogenous melatonin pretreatment did not apparently change the phenotypes of kiwifruit plants under control condition. While the waterlogging treatment resulted in visible leaf damage on kiwifruit plants in both WL and MT+WL groups, the melatonin pretreatment definitely alleviated the stress symptoms, which was indicated by decreased electrolyte leakage and MDA levels. Previous studies have reported that melatonin application could improve the photosynthesis rate and PSII efficiency in apple (Zheng et al., 2017) and alfalfa (Zhang et al., 2019) to alleviate the stress damage during waterlogging treatment. Here, the decrease in Pn of kiwifruit plants caused by waterlogging stress was also effectively lightened by melatonin pretreatment. The substantial increase in the Ci value of WL plants also indicated substantial disorders in the leaf photosynthetic structure of non-pretreated plants under waterlogging stress.

Since the plant root is the front line to continuously sense waterlogging signal, we performed further researches on kiwifruit roots. In addition to the root activity mentioned earlier in the concentration screening test, the detected relative electronic leakage and MDA concentration of the roots also showed milder increment in the kiwifruit plants with exogenous melatonin pretreatment, which indicated that exogenous melatonin could protect the integrity and vitality of the root system under waterlogging stress, thereby reducing waterlogging damage to the kiwifruit plants. Moreover, the measurements showed that both the ROS generation and antioxidant enzymes activity were induced by waterlogging stress in kiwifruit root, and the melatonin-treated plants possessed a higher antioxidant ability and lower ROS accumulation. As these results were consistent with previous researches, which reported that melatonin has important functions in scavenging ROS in different plant species in response to various biotic and abiotic stresses (Li et al., 2018; Liang et al., 2019), we believed that the melatonin treatment enhanced the waterlogging tolerance of kiwifruit plants through regulating the ROS pathway in roots.

The re-orchestration of plant primary metabolism, including the amino acid biosynthesis, plays a major role in the metabolic adjustment of plants under waterlogging stress (Batista-Silva et al., 2019; Behr et al., 2021). Moreover, previous studies have found that compared with the stronger responses of plant roots to waterlogging conditions, the hypoxic symptoms of shoots seem to be less, which probably due to the oxygen generated by leaf photosynthesis (Mustroph et al., 2014). Here, we also paid more attention on the changes in the amino acid metabolism of kiwifruit roots during waterlogging treatment. The detection showed a large increment of Ala and GABA concentrations in kiwifruit roots during waterlogging period, which were reported as a marker of hypoxic symptoms in other plant species (Rocha et al., 2010; Oliveira and Sodek, 2013), and the melatonin treatment promoted the increase of GABA while reduced the accumulation of Ala. Furthermore, we also detected a significant decrease in Asp and Glu concentrations in kiwifruit roots during waterlogging treatment, which was enhanced by melatonin treatment. The decrease in Asp and Glu levels caused by hypoxia has been reported in *Arabidopsis* and other plant species (Gibon et al., 2002; Mustroph et al., 2014), consistent with the role of these two amino acids as precursors for the synthesis of Ala and GABA. Since the reaction from Glu to GABA consumes protons, previous studies proposed the buffer role of GABA to counterbalance the detrimental effects of cellular acidification during hypoxia (Carroll et al., 1994). Here, our results showed that melatonin treatment facilitated the utilization of Asp and Glu in kiwifruit roots under waterlogging stress, and promoted the transition to GABA than to Ala. Combined with previous research which reported that the submergence intolerant variety of rice showed a higher elevation of Ala levels than tolerant variety under submerged condition (Barding et al., 2013), we believed that melatonin enhanced the waterlogging tolerance of kiwifruit plants partly through altering the Ala and GABA metabolism.

Synthesis of Pro and BCAAs and degradation of Arg in kiwifruit roots was enhanced by melatonin treatment during waterlogging stress. The Pro has been widely suggested to be indispensable for plant response to environmental stresses (Antoniou et al., 2017; Huo et al., 2022), and it was reported to be involved in the redox buffering system of the plant cells during hypoxia (Behr et al., 2017). The high-level induction of BCAAs was reported in plants in response to various stress conditions (Huang and Jander, 2017). Here, we found that the metabolism of Pro and BCAAs in kiwifruit roots was enhanced by melatonin treatment to encounter waterlogging stress. As Arg serves as a substrate for polyamines (PAs) and nitric oxide, which was proved to be favorable in plant waterlogging tolerance (Pucciariello and Perata, 2017; Rauf et al., 2021), melatonin might promote the waterlogging resistance of kiwifruit plants by regulating the Arg transformation. Previous researches in alfalfa have reported that melatonin could modulate the nitro-oxidative homeostasis and proline metabolism to ameliorate drought damage (Antoniou et al., 2017), but the detailed interaction mechanisms between melatonin, PAs and nitric oxide in plants in response to waterlogging stress require further study. Besides the amino acid levels, we also analyzed the changes in the ACC, ABA, and SA levels in kiwifruit roots under waterlogging stress. Previous researches reported that the concentration of ABA and SA changed in soybean hypocotyls under waterlogging stress, which might contribute to the secondary aeration tissues appearing (Shimamura et al., 2014; Kim et al., 2015). Moreover, Qi et al. (2019) found that treating cucumber seedlings with exogenous ACC promoted the formation of adventitious roots under waterlogging stress. However, the relationship among melatonin and them when plants encountered waterlogging stress were rarely reported. Here, the detection showed that while the ACC concentration in kiwifruit roots was induced markedly by waterlogging stress, the ABA and SA concentrations decreased. Apparently, the melatonin treatment enhanced the ACC increment in kiwifruit roots under waterlogging stress. ACC is the direct precursor in the biosynthesis of ethylene, and the ethylene accumulation and perception are necessary for aerenchyma cells formation and adventitious root primordium generation (Yamauchi et al., 2014; Yamauchi et al., 2016). The enhanced ACC levels in kiwifruit roots under waterlogging stress might contribute, to a certain extent, in melatonin-mediated waterlogging tolerance.

## Conclusion

In conclusion, our results demonstrated that pre-irrigating kiwifruit plants with 100 μM melatonin could partially alleviate the waterlogging damage on whole plant. First, the kiwifruit plants in MT+WL group maintained a better photosynthetic activity during the treatment, and they showed a less ROS accumulation in roots after waterlogging stress. Further study on kiwifruit roots showed that the levels of Ala, GABA, and Pro were increased markedly due to waterlogging stress, and melatonin treatment promoted the increment of GABA and Pro while reduced the accumulation of Ala. These responses suggested that melatonin-mediated waterlogging tolerance of kiwifruit plants was related to Ala, GABA, and Pro metabolism. Moreover, melatonin pretreatment altered the level of other plant hormones in kiwifruit roots to encounter waterlogging stress, which included the enhancement of ACC accumulation. The present study provided novel insights into the melatonin-induced waterlogging tolerance of kiwifruit plants, as well as the new and initial evidence for the effect of exogenous melatonin on plant amino acid metabolism. The specific and detailed interaction mechanisms between melatonin and metabolic pathways in the plant-water interactive system require further study.

## Data availability statement

The raw data supporting the conclusions of this article will be made available by the authors, without undue reservation.

## Author contributions

LH and KX designed the experiments. LH, HW, and QW performed the experiments and analyzed the data, assisted by YG. LH and XS wrote and revised the manuscript. All authors contributed to the article and approved the submitted version.
